# Luciferase-induced immune activation prevents orthotopic K7M2luc osteosarcoma establishment in BALB/c mice

**DOI:** 10.2478/raon-2026-0049

**Published:** 2026-09-07

**Authors:** Sasa Kupcic, Urska Kamensek, Maja Cemazar, Ursa Lampreht Tratar

**Affiliations:** 1 Department of Experimental Oncology, Institute of Oncology, Ljubljana, Slovenia; 2 Faculty of Medicine, University of Ljubljana, Ljubljana, Slovenia; 3 Biotechnical Faculty, University of Ljubljana, Ljubljana, Slovenia; 4 Faculty of Health Sciences, University of Primorska, Izola, Slovenia; 5 Veterinary Faculty, University of Ljubljana, Ljubljana, Slovenia

**Keywords:** mouse orthotopic tumor, luciferase, bioluminescence, osteosarcoma

## Abstract

**Background:**

Use of murine orthotopic tumor models dictates utilization of luciferase-expressing cells for *in vivo* bioluminescence imaging. However, luciferase may trigger immune responses that impair tumor establishment.

**Materials and methods:**

We transduced K7M2 cells wild-type (wt) with luciferase gene and evaluated their growth *in vitro*. We then implanted cells orthotopically in BALB/c mice and upon unsuccessful tumor establishment assessed systemical and local mechanism for tumor growth suppression.

**Results:**

All investigated cell lines had a comparative doubling time *in vitro* at 24 and 48h. Despite that, K7M2luc E5 differed from K7M2luc A7 and K7M2 wt by having a much higher proliferation rate at 72h (35x, 15x and 17x). *In vivo*, both luciferase-expressing cell lines failed to establish tumors, whereas K7M2 wt had a 100% success rate. To investigate the underlying mechanism, we assessed systemic and local cell-mediated immunity. Mice in groups K7M2luc A7 and K7M2luc E5 had a higher fold change (5 ± 0.8 and 6 ± 1.05) of GrB secreting splenocytes than naïve mice (1 ± 0.2). In the bone microenvironment, luciferase groups exhibited elevated numbers of GrB-secreting cells and CD8^+^ T cells relative to K7M2 wt and naïve mice.

**Conclusions:**

These findings indicate that luciferase activates cytotoxic T-cell responses capable of eliminating implanted cells. Tumor establishment was restored in immunodeficient mice lacking CD8^+^ cells, confirming the immunemediated rejection.

## Introduction

Osteosarcoma is a rare, highly aggressive primary malignant bone tumor that predominantly affects adolescents and young adults. It accounts for approximately 2.4% of all pediatric malignancies.^1,2^ Most frequently it arises in metaphyseal regions of long bones, particularly the distal femur, proximal tibia, and proximal humerus.^3^ Histopathologically, osteosarcoma is characterized by malignant mesenchymal cells that undergo aberrant osteoblastic differentiation, leading to the production of immature and structurally fragile bone matrix, termed osteoid.^4,5^

Bone microenvironment is a highly complex and dynamic system composed of bone, stromal, endothelial, and immune cells that are closely integrated with the mineralized extracellular matrix.^6^ Tumor cells establish intensive communication with this environment through a wide array of cytokines, chemokines, and growth factors, thereby promoting the development of a favorable tumor microenvironment (TME).^7^ Given the unique characteristics of the bone niche in which sarcomas develop, use of mouse orthotopic models is strongly recommended and surpasses the use of subcutaneous tumors.^8,9^ Their growth and invasiveness are more representative of the clinical course of the disease in patients. Evidence from studies of colorectal, bladder, prostate, and pancreatic cancer have demonstrated that cancer cells implanted in orthotopic sites can acquire a metastatic phenotype, underscoring the importance of these models for investigating the metastatic process.^10-13^ Moreover, tumor implantation site can significantly influence antitumor efficacy of experimental therapies. For example, in a study comparing colorectal carcinoma cells implanted subcutaneously versus orthotopically, inhibition of tumor growth following doxorubicin treatment was twice as pronounced in subcutaneous tumors compared with orthotopic tumors.^14^

Orthotopic localization of osteosarcoma tumors poses considerable challenges for their longitudinal monitoring and growth assessment. Non-invasive bioluminescence imaging (BLI) is a widely adopted approach for pre-clinical evaluation. This technique relies on the stable transduction of tumor cell lines with a bioluminescent reporter gene prior to their inoculation into mice.^15^ One of the most widely used BLI markers is luciferase (luc), mainly due to its noninvasiveness, sensitivity and affordability. Luciferases are animal-derived enzymes that produce light in presence of an appropriate substrate. Luciferases differ among themselves according to the animal of origin, e.g. sea pen (Renilla luciferase), black prince copepod (Gaussia luciferase) or the most used firefly luciferase, which derives from the common eastern firefly (*Photinus pyralis*).^16^ For imaging, animals are administered the substrate (e.g. D-luciferin), typically via intraperitoneal or subcutaneous injection.^17^ *In vivo* imaging system (IVIS) subsequently detects and quantifies emitted bioluminescent signal, which is a result of luciferin oxidation due to presence of luciferase enzyme. This method enables sensitive, real-time visualization and quantification of tumor burden without the need for invasive procedures.^18,19^

Despite the widespread use of BLI, some studies utilizing luciferase-expressing cell lines report a delay in tumor growth and subsequent reduced metastatic potential. Such examples include a pancreatic ductal adenocarcinoma model, where a cell line with incorporated red-shifted firefly luciferase failed to establish an orthotopic tumor, whereas a cell line transduced with a click-beetle green luciferase proceeded to establish tumors. Other groups reported similar problems with a firefly luciferase in a glioblastoma tumor model.^20,21^ Luciferase can reportedly suppress tumor growth even in cases of subcutaneous implantation, as was reported by Brutkiewicz *et al*., where they demonstrated that in case of subcutaneous implantation luciferase expression levels directly altered *in vivo* tumor growth dynamics, underscoring biological effects beyond imaging.^22^ Luciferase has even been deliberately employed as a model immunogen in DNA immunization study, highlighting its intrinsic immunostimulatory potential.^23^ In our own earlier work, we also demonstrated that reporter luciferase expression can contribute to antitumor effects.^24^ Stated interferences in tumor development can be attributed to the immune response, spearheaded by the expression of intracellular luciferase.^20,22,25^

Codon-optimized and improved firefly luciferase, named luc2, is widely utilized and even though it was shown to be successful in establishing K7M2luc osteosarcoma tumors in relatively small number of syngeneic BALB/c mice, there are several studies on 4T1 mammary tumors and glioblastoma tumors, where luc2 cell lines showed to be detrimental to tumor growth.^25-28^

However, numerous studies, that also described luciferase-based imaging modalities in immunocompetent mice, did not report any detectable immunogenic limitations.^28-30^ Furthermore, some reports specifically evaluated the potential adverse effects of luciferase expression on tumor growth and found no evidence of a detrimental impact.^31^

Conflicting or limited evidence has likely contributed to the immunogenicity of reporter genes being underestimated, resulting in this phenomenon remaining insufficiently addressed in routine experimental practice.

Therefore, our aim was to investigate the interference of luc2 with development of murine orthotopic osteosarcoma models in immunocompetent mice which has not been reported yet.

## Materials and methods

### Establishment of transduced cell line

Murine osteosarcoma (OS) cell line K7M2 wild type (wt) (CRL-28369) was obtained from the American Type Culture Collection (ATCC; Manassas, VA, USA). Cells were cultured in Advanced Dulbecco’s Modified Eagle Medium (Advanced DMEM; Gibco, Thermo Fisher Scientific, Waltham, MA, USA), supplemented with 5% (v/v) fetal bovine serum (FBS; Gibco), 100 U/mL penicillin, 100 μg/mL streptomycin (Sigma-Aldrich, Darmstadt, Germany), and 1% (v/v) GlutaMAX (Gibco), in a humidified incubator at 37 °C with 5% CO_2_. Mycoplasma contamination was routinely monitored using the MycoAlert™ PLUS Mycoplasma Detection Kit (Lonza Group Ltd., Basel, Switzerland), and all cultures were confirmed to be mycoplasma-free.

The transduced cell lines were established using two different modalities, with in-house preparation of lentivirus particles and by using commercial lentiviral particles.

#### Plasmid construction and lentiviral transduction

K7M2luc A7 cell line was generated via lentiviral transduction as previously described.^32^ Luciferase transfer plasmid was constructed by replacing the enhanced green fluorescent protein (eGFP) gene in pLenti PGK GFP Puro plasmid (w509-5; a gift from Eric Campeau and Paul Kaufman, Addgene plasmid #19070) with luciferase 2 (*Photinus pyralis*) (Luc2 CO) gene fragment (gBlocks™ HiFi Gene Fragments; Integrated DNA Technologies, Coralville, IA, USA). Luc2 CO derived from luc2 gene fragment that was further codon-optimized for mouse expression by the tool for codon optimization (Integrated DNA Technologies). Lentiviral packaging was achieved using plasmids pMDLg/pRRE, pMD2.G, and pRSV-Rev (gifts from Didier Trono; Addgene plasmids #12259, #12251, and #12253, respectively).

All four plasmids were propagated in *Escherichia coli* under ampicillin selection and isolated per manufacturer’s instructions, using EndoFree Plasmid Mega Kit (Qiagen, Hilden, Germany). Lentiviral particles were produced in 293T cells (CRL-3216, ATCC) grown to 80% confluence in 6-well plates. Modified transfer plasmid was co-transfected with packaging plasmids pMDLg/pRRE, pMD2.G, and pRSV-Rev using Lipofectamine 2000 (Invitrogen, Thermo Fisher Scientific). At 24 hours post-transfection, medium was replaced with 5 mL of fresh complete medium. Supernatants containing viral particles were harvested 48 and 72 hours after transfection, pooled, centrifuged at 500 × g for 10 minutes, and filtered through a 0.45 μm membrane (Merck Millipore, Burlington, MA, USA).

For transduction, K7M2 wt cells were seeded in 6-well plates and grown to 80% confluence. Culture medium was replaced with afore mentioned viruscontaining medium with added polybrene (4 μg/mL; Sigma-Aldrich) and incubated for 24 hours. At 48 hours post-transduction, puromycin (8 μg/mL; Sigma-Aldrich) was added for selection. A monoclonal stable cell line was established by seeding single cells into 96-well plates and maintaining puromycin selection. After 10 days, colonies were harvested and transferred to T75 flasks. Once confluence was achieved in T75 flasks, monoclonal populations were generated and screened for bioluminescent intensity with IVIS Lumina XMRS (Revvity, Waltham, MA, USA). Clone displaying a sufficient bioluminescent intensity was selected (clone K7M2luc A7), expanded under standard conditions with continuous puromycin selection, and stored in liquid nitrogen.

#### Transduction with commercially available viral particles

K7M2luc E5 cell line was established using IVISbrite Red F-luc-Puromycin Lentiviral Particles (RediFect; Revvity) according to manufacturer’s instructions. K7M2 wt cells were seeded into 24-well plates and grown to 80% confluence. Cells were transduced at varying multiplicities of infection (MOIs) in presence of polybrene (4 μg/mL) to enhance transduction efficiency. After 24 hours, medium was replaced, and puromycin (8 μg/mL) was added after another 24 hours for selection. Cells were then seeded to 96-well plates to establish monoclonal populations which were then transferred to T75 flasks. Upon reaching confluence in T75 flasks, clone exhibiting a sufficient bioluminescent intensity was selected (clone K7M2luc E5), expanded under standard conditions with continuous puromycin selection and cryopreserved in liquid nitrogen.

### Cell proliferation assay and luciferase expression *in vitro*

For assessment of cell growth kinetics K7M2 wt, K7M2luc E5 and K7M2luc A7 cells were seeded to a 96-well plate (1.5x10^3^ cells/well). Fluorescence based assay with Presto Blue™ reagent (Thermo Fischer) was performed. At different time points (0, 24, 48 and 72 h) 10 μL of the reagent was added to wells. Upon 1 h incubation in a CO_2_ incubator, fluorescence of a metabolic product resorufin was measured at 530/590 ex/em with a multimodal reader Cytation 1 (BioTek, Winooski, VT, USA).

Luciferase expression *in vitro* was determined by seeding 100 μl of a cell suspension (concentration of 1x10^5^cells/ml) to a 96-well plate. D-luciferin (30 mg/mL; Thermo Fischer) was added to wells with cell suspensions at different working concentrations. Plates were then incubated for 10 min in a CO_2_ incubator and imaged using imaging system IVIS Lumina XMRS.

### Animals and tumor induction

6–8 weeks old female 9 BALB/c and 6 NUDE mice from Charles River Laboratories (Lecco, Italy) were used. Animals were housed in cages of 3–6 animals per cage in specific-pathogen-free conditions in a carousel mouse IVC rack system (Animal Care Systems Inc., Revere Parkway, USA) at a temperature of 20–24°C, 55 ± 10% relative humidity, 12 h light/dark cycle, food and water ad libitum and cage enrichment. For tumor induction in BALB/c mice, 1 × 10^6^ K7M2 wt, K7M2luc A7 or K7M2luc E5 cells were resuspended in 25 μL of saline solution. Mice were placed under isoflurane anesthesia (2–3%) and cell suspension was injected intratibially to the shaved proximal site of the anterolateral region of right hind leg. Further on in the animal experiment, confirming the role of immune cells in NUDE mice, the mice were inoculated with 1 × 10^6^ of only K7M2luc A7 cell line.^26^ This cell line, which demonstrated lower luciferase expression compared to K7M2luc E5, was chosen based on previous reports showing that cell lines with higher luciferase expression exhibited reduced tumor growth as opposed to similarly produced cell clones with a lower luciferase expression.^22^ Buprenorphine (0.1 mg/kg body weight, 0.015 mg/mL) was administered perioperatively and 8 hours postoperatively. Animals were monitored daily for signs of distress and weighed twice a week. All procedures were planned and performed according to PREPARE, ARRIVE and OBSERVE guidelines and approved by the Ministry of Agriculture, Forestry, and Food of the Republic of Slovenia (permit no. U34401-17/2023/1).

### Bioluminescence imaging and X-ray imaging

Tumor progression was monitored twice a week using BLI and once a week by X-ray imaging. Substrate D-luciferin (15 mg/mL) (GoldBio, St. Louis, MO, USA) was administered intraperitoneally at a dose of 150 mg/kg body weight. Mice were placed under isoflurane anesthesia (2–3%) and images of the lower half of the body were captured by IVIS Lumina XRMS (Revvity) 15 minutes after D-luciferin administration. Exposure was set to automatic with a maximum time of 2 minutes, binning was set to 8. Images were analyzed with Living Image software (Revvity); regions of interest (ROIs) were drawn over tumor sites, and total photon flux (photons/sec) was quantified after background subtraction.

In all groups, X-ray imaging was utilized once a week as previously mentioned. Mice were placed under isoflurane anesthesia (2–3%), followed by imaging of the inoculated knee joint. Each image was recorded with a 96-s exposure, tube potential 35 kVp and a tube current of 100 μA. Binning was set to 1.

### Sample collection

When bioluminescence signal in BALB/c mice dropped below detection threshold (1 × 10^4^ photons/second), mice were placed under anesthesia and blood was collected from the retro-orbital venous sinus and centrifuged (20 min, 2000 × g) to obtain a serum, which was then frozen at –80 °C. Mice were humanely euthanized by a method of cervical dislocation. Spleens were aseptically harvested, mechanically sheared, and strained through a sterile 50 mm strainer to obtain single-cell splenocyte suspension. Cells were centrifuged (5 min, 500 × g, 4°C), washed with chilled PBS and resuspended in chilled red blood cell lysis buffer for 5 min. Reaction was stopped by 4 × dilution with chilled PBS. After centrifugation, pellets were resuspended in chilled serum-free freezing medium (Bulldog Bio, Portsmouth, NH, USA) and frozen at –80 °C.

Tibias, previously inoculated with tumor cells, were removed, fixed in 10% neutral buffered formalin and decalcified in EDTA (MoL-DECALCIFIER, Milestone, Valbrembo, ITA). Upon decalcification, samples were embedded in paraffin and cut in 2 μm thick sections for H&E or immunohistochemical staining. Tissue sections, stained with H&E, were evaluated for necrotic and viable areas, indicated by pink and purple staining, respectively. Samples were also collected from naïve mice without any prior tumor inoculation.

NUDE mice were split into groups for tumor establishment (2 mice) and tumor growth observation (4 mice). Mice from the first group were euthanized at the time of tumor establishment and tumor samples were collected for immunohistochemical staining. Second group was observed with BLI and X-ray imaging continuously until signal strength started faltering or animals started to exhibit signs of clinical distress (lack of grooming, minimal loss of weight, limping…), at which point they were humanely euthanized with cervical dislocation.

### Immunohistochemistry staining

2 μm thick paraffin embedded tissue sections were marked using 2 different primary antibodies: rabbit anti-mouse anti-CD8 alpha Ab (ab209775; Abcam, Danaher, Washington, D.C., DC, USA) and rabbit anti-mouse anti-Granzyme B Ab (ab4059, Abcam). Tissue sections were first incubated at 60° C for an hour, then deparaffinization and antigen retrieval (20 min, 97°C) were performed in a PT Link Pre-Treatment module for Tissue Specimens (Agilent Technologies, Santa Clara, Ca, USA) with a low pH Target Retrieval Solution EnVision FLEX (Dako Omnis, Agilent). Sections were subsequently incubated in a humid chamber with primary antibodies overnight at 4°C. Dilutions of primary antibodies in blocking buffer were as follows: 1/1000 for anti-CD8 and 1/1000 for anti-Granzyme B. Subsequently, biotinylated secondary antibodies (goat anti-rabbit IgG) (Abcam) were added, followed by a chromogenic reaction with rabbit specific HRP/DAB (ABC) detection IHC Kit (ab64261; Abcam) according to manufacturer’s instructions. Slides were dried overnight and imaged with NanoZoomer S360MD digital slide scanner (Hamamatsu Photonics, Hamamatsu, Japan).

To quantify infiltration of CD8^+^ cells and secretion of granzyme B (GrB), ten high-power fields (HPFs) were selected, representing a total area of 1 mm^2^ of the entire scanned slide. The number of positively stained objects within each HPF was then counted in blind fashion by 2 independent examiners.

### ELISpot GrB assay

An ELISpot assay was performed to assess cytotoxic lymphocyte activity via GrB secretion. Splenocytes were thawed overnight and activated with a luciferase peptide H-GFQSMYTFV-OH (20μg/mL, 20h; Biosynth Ltd., Berkshire, UK), luc2-specific CD8^+^ epitope in BALB/c mice, that was previously described as being used to activate splenocytes in *ex vivo* studies.^25,33-37^ Splenocytes were then seeded to ELISpot plate (5 × 10^4^ cells/well), followed by 48 h incubation. ELISpot assay was then performed according to the manufacturer’s instructions. GrB spot-forming cells were counted using a stereomicroscope Zeiss Lumar.V12 (Zeiss, Oberkochen, Germany). Results were normalized to unactivated splenocytes.

### ELISA interferon-γ assay

To detect the presence of interferon-γ (IFN-γ), mice serum was thawed and added to the ELISA plate (R&D systems, Minneapolis, MN, USA) along with reagents according to the manufacturer’s instructions. Optical density was determined with Cytation 1 multimodal plate reader set to 450 nm, with wavelength correction at 570 nm.

To collect cell culture supernatant, splenocytes were thawed and allowed to recuperate for 24 h in a humidified CO_2_ incubator. They were reactivated with a luciferase peptide H-GFQSMYTFV-OH (20 μg/mL, 20 h; Biosynth Ltd., Berkshire, UK) and added to a plate with previously seeded cells at a target:effector ratio of 1:10 and left to incubate for 48 h in a CO_2_ incubator. Supernatant was removed, centrifuged to remove cell debris and concentrated 2.5x times in centrifuge tubes with a centrifugal filter (Millipore). 50 μl of each sample was then added to the ELISA plate (R&D systems, Minneapolis, MN, USA) along with reagents according to the manufacturer’s instructions. Optical density was determined with Cytation 1 multimodal plate reader set to 450 nm, with wavelength correction at 570 nm.

### Statistical analysis

Statistical analysis and graphing were done in GraphPad Prism software 10.4.2 (Graphpad Software, Boston, MA, USA). Results were first tested for normal distribution using the Shapiro-Wilk or D’Agostino-Pearson test. One-way analysis of variance (ANOVA), Student’s t-test and Welch’s t test were used to determine differences among experimental groups, where data was normally distributed. Differences were considered statistically significant if the p value was below 0.05. Data are presented as arithmetic means (AM) and standard errors of means (SEM).

## Results

### In vitro evaluation of cell proliferation and luminescence signal

**FIGURE 1.**
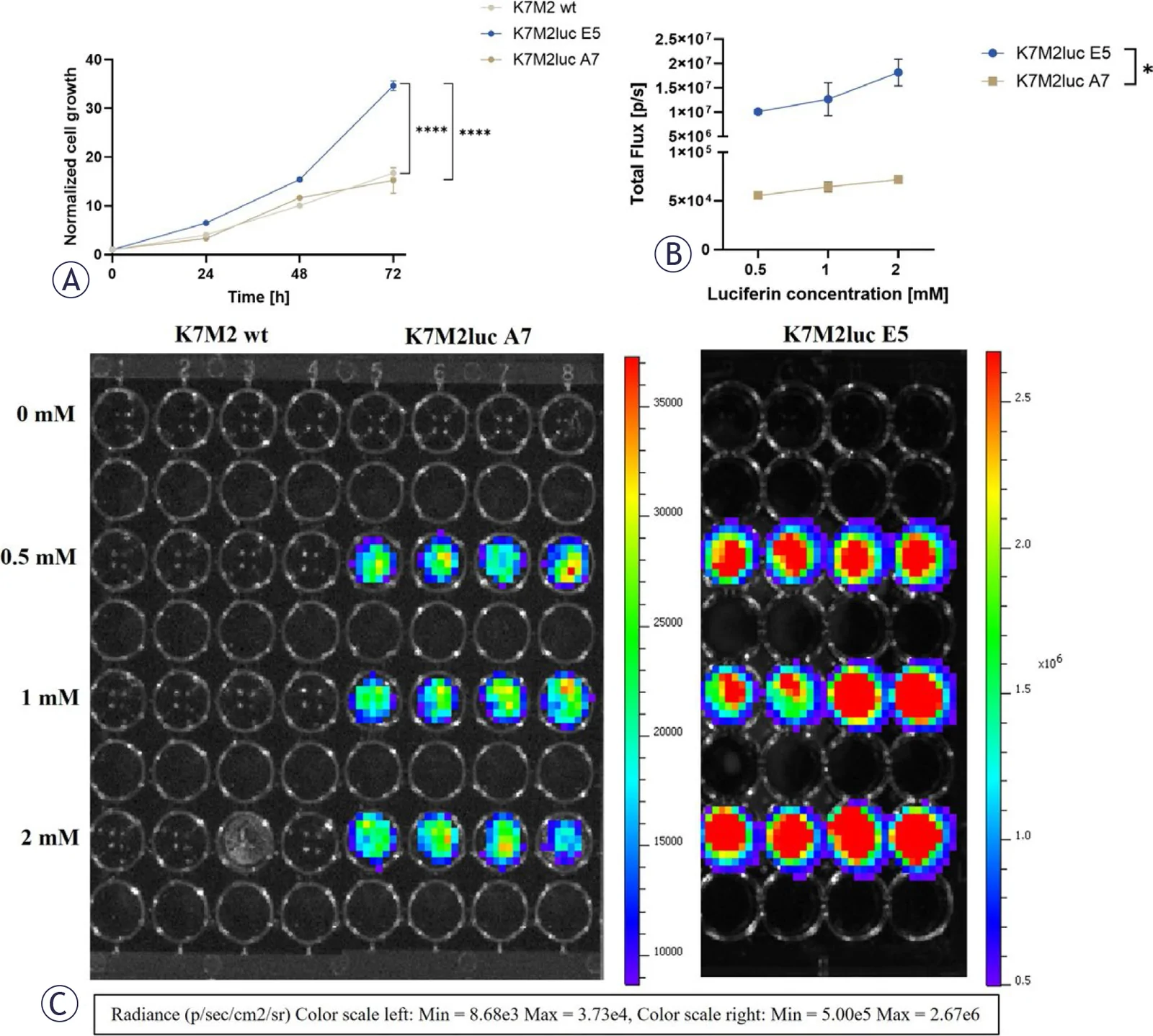
**(A)** All cell lines show exponential growth during time points 24 h, 48 h and 72 h. **(B)** K7M2luc A7 and K7M2luc E5 clonal lines show no dose-dependent statistically significant differences in bioluminescent signal, K7M2luc E5 has a higher bioluminescent signal at all added luciferin doses. **(C)** BLI of cell lines with added luciferin (0 –2 mM). n = 4–12. Values are presented as Arithmetic Mean (AM) ± Standard Error of the Mean (SEM). * p < 0.01, **** p < 0.0001. Where not indicated with *, differences are not significant. Paired t-test, one-way ANOVA.

We assessed the effect of luciferase expression on cell proliferation kinetics with a fluorescence-based assay Presto Blue™. All cell lines exhibited exponential growth during time points of measurement, with similar proliferation rates at time points 24 h (3–6×) and 48 h (10–15×) (Figure 1A). However, at time point 72 h, K7M2luc E5 cell line had the highest proliferation rate, 35×, which significantly differed from proliferation rates of cell lines K7M2luc A7 and K7M2 wt, which were 15× and 17× respectively (p < 0.0001). From the measured data, doubling times for each cell line were also calculated. K7M2luc E5 had a doubling time of 19 h, K7M2luc A7 26 h, and K7M2 wt 25 h. Differences in doubling times were not statistically significant.

Expression of luciferase was assessed by adding varying concentrations of D-luciferin substrate (0 mM–2 mM) to K7M2luc clonal lines, with K7M2 wt cells serving as a negative control. No dosedependent differences in bioluminescent signal intensity were observed across substrate concentrations (Figure 1B). However, statistically significant differences were detected among the luciferaseexpressing clones. K7M2luc E5 cell line exhibited robust bioluminescence, with signal intensities ranging from 6.5 × 10^6^ to 2.3 × 10^7^ photons/second (p/s), whereas K7M2luc A7 demonstrated markedly lower levels of signal, averaging approximately 6 × 10^4^ p/s (p = 0.0294, Figure 1C).

### Evaluation of K7M2 wt, K7M2luc A7 and K7M2luc E5 growth rate after tumor inoculation in BALB/c mice

**FIGURE 2.**
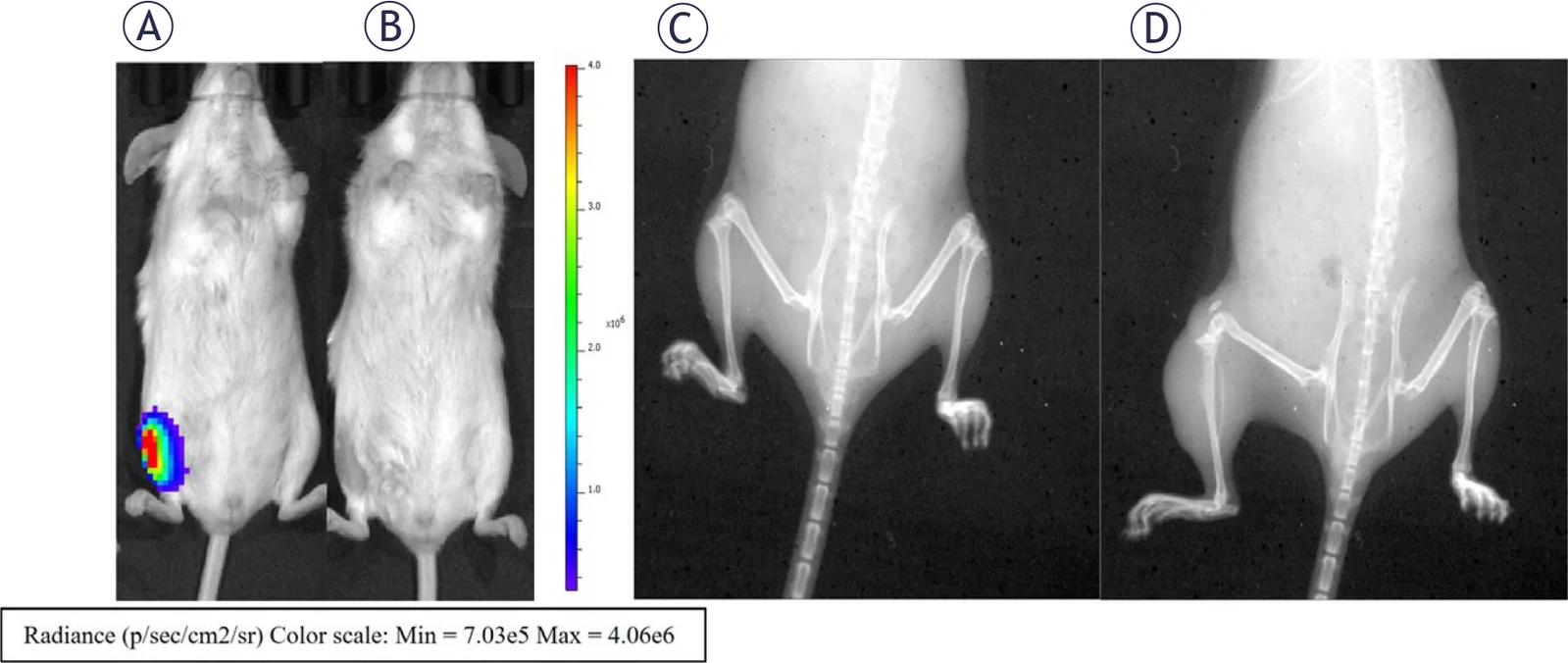
Representative images of BLI and X-ray imaging. BALB/c mice, inoculated with K7M2luc A7 at **(A)** day 6 and **(B)** day 8 demonstrating a decrease in signal. BALB/c mice, inoculated with K7M2 wt at **(C)** day 6 and **(D)** day 25 demonstrating progressive tumor growth due to bone lysis.

*In vivo* tumor growth was examined by first implanting K7M2 wt or luciferase-expressing clonal lines to the mice tibia. To observe tumor growth, K7M2 wt group was imaged once a week using X-ray imaging, whereas K7M2luc A7 and E5 mice were imaged twice a week using BLI and X-ray once a week (Figure 2A–D).

**FIGURE 3.**
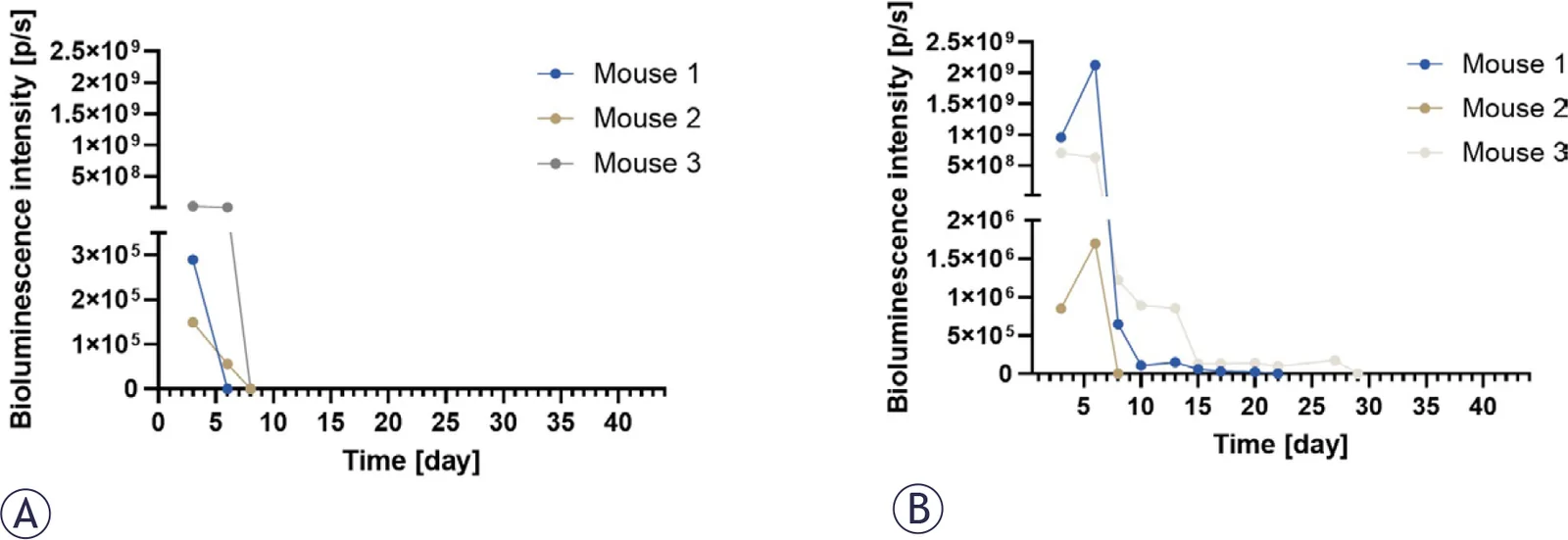
**(A)** BALB/c mice, inoculated with K7M2luc A7, showed a strong signal on day 3 with a drastic drop on day 6. **(B)** K7M2luc E5-inoculated BALB/c mice had an increase in signal strength on day 6 and a delayed fall day 8–29. n = 3. Values are presented as Arithmetic Mean (AM) ± Standard Error of the Mean (SEM).

K7M2luc A7 group had a strong positive signal on day 3 post inoculation, from 1.5 × 10^5^ to 2.2 × 10^7^ p/s, with a prominent decline in signal strength to 0 p/s on day 6 (Mouse 1) and day 8 (Mouse 2, 3) (Figure 3A), when mice were sacrificed and samples were collected. K7M2luc E5 group also had a positive signal at day 3, between 8.5 × 10^5^ and 9.5 × 10^8^ p/s. Unlike K7M2 A7 group, there was a general increase in signals on day 6 with a subsequent fall (Figure 3B). Mice were sacrificed and samples were taken on day 8 (Mouse 2), day 22 (Mouse 1) and 29 (Mouse 3), when the bioluminescence signal was under 1 × 10^4^ p/s. K7M2 wt group was observed for 25 days, with tumor detection due to bone lysis and soft tissue growth at the site of cell inoculation in all three mice (Figure 2D). On day 25, mice were sacrificed, and samples were collected for continuation of studies.

### Assaying activation of cytotoxic effector cells in mice on systemic and local level

**FIGURE 4.**
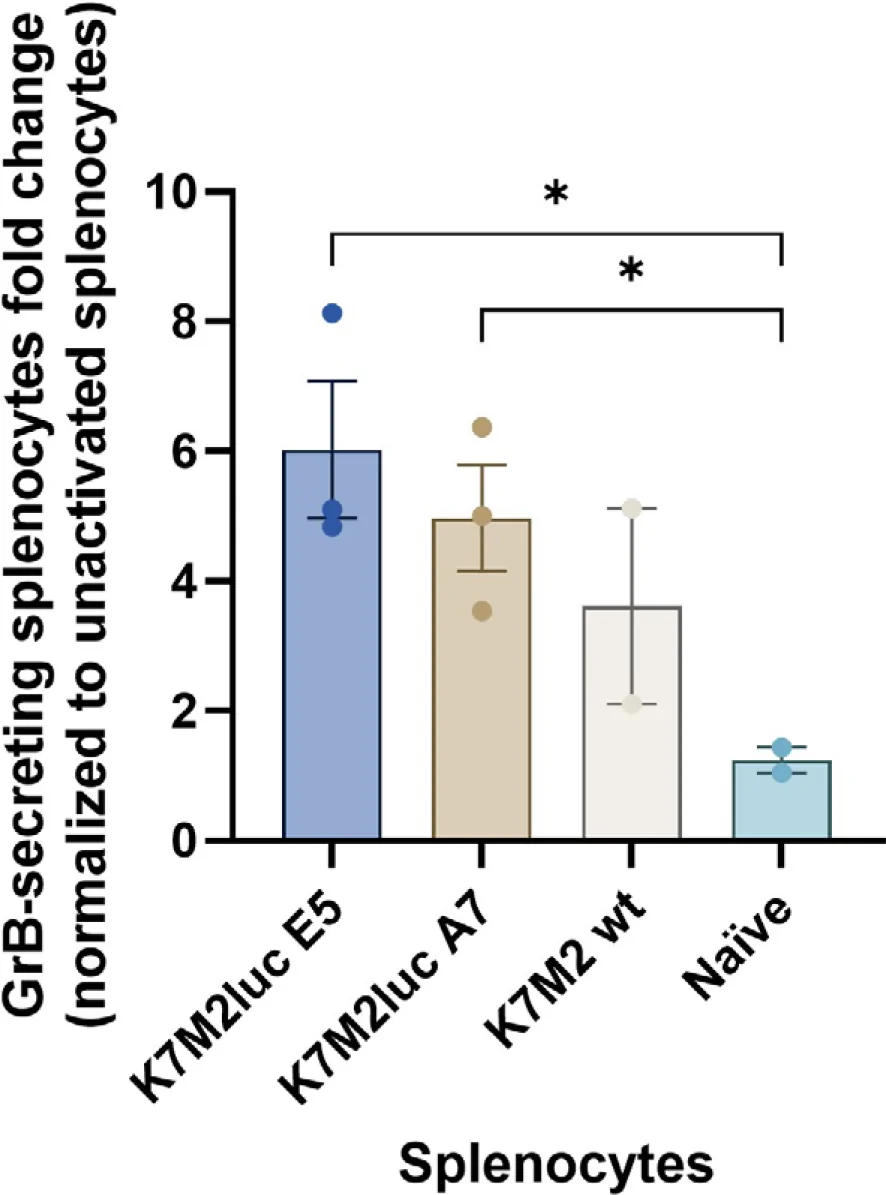
GrB positive splenocytes from inoculated mice with unsuccessful tumor growth. Splenocytes underwent secondary activation with luciferin peptide. n = 2–3. Values are presented as Arithmetic Mean (AM) ± Standard Error of the Mean (SEM). * p < 0.05. Where not indicated with *, differences are not significant. Welch’s t test.

Activation of cytotoxic effector cells (CD8^+^ T cells and NK cells) in response to immunogenic properties of luciferase protein was assessed by measuring the amount of GrB-secreting splenocytes by a quantitative ELISpot assay. The highest fold change in GrB-secreting splenocytes was detected in mice inoculated with luciferase-expressing cell lines, with values of 6 ± 1.05 (K7M2luc E5) and 5 ± 0.8 (K7M2luc A7). Lower numbers of GrB-secreting splenocytes were detected in splenocytes isolated from naïve mice with a fold change of 1 ± 0.2. Activation of examined immune cells in mice with K7M2 wt inoculation was lower than in those inoculated with luciferase-expressing cell lines (Figure 4). Statistical analysis indicated differences among groups K7M2luc E5, K7M2luc A7 and Naïve (p = 0.04), although the low number of biological replicates limits statistical robustness.

ELISA for secretion of IFN-γ was performed on both serum samples and spleen-derived supernatant samples. No detectable IFN-γ was observed in either the serum or supernatant samples. Secondary activation of splenocytes *in vitro* with the luciferase peptide, as well as subsequent concentrating of supernatant samples, did not yield measurable IFN-γ levels. These findings indicate that IFN-γ production was either absent or below the detection threshold of the assay under tested conditions.

### Immunohistochemistry for observation of immune cell infiltration to the tumor site

**FIGURE 5.**
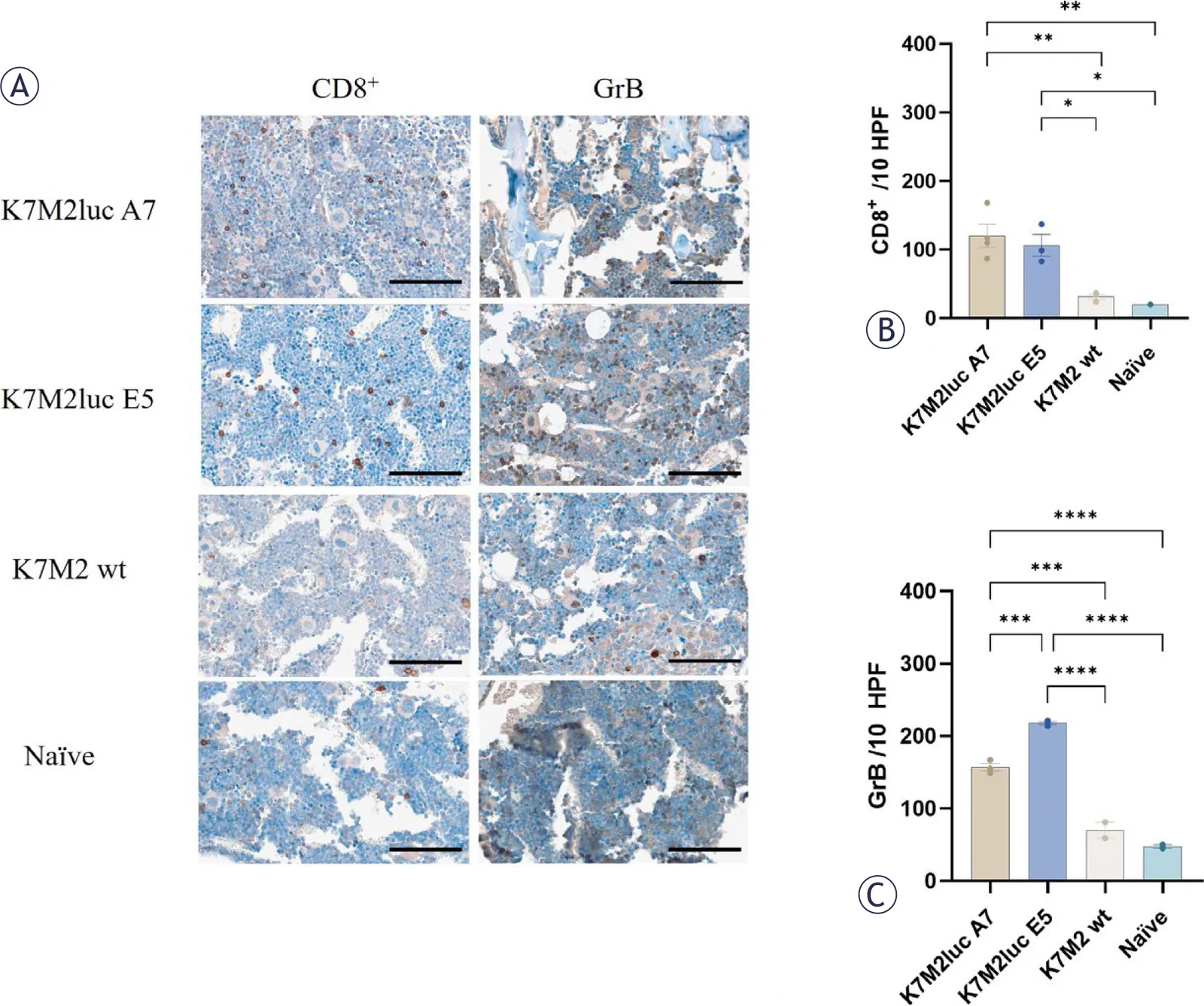
**(A)** Representative images of tibial paraffin sections collected after the bioluminescence signal dropped below the detection threshold and stained with anti-CD8 and anti-GrB antibodies. 40x magnification, scale bar = 100 μm. **(B)** CD8^+^ positive cells/10 high-perfusion fields (HPF). **(C)** GrB positive cells/10 (HPF). n = 2–3. Values are presented as Arithmetic Mean (AM) ± Standard Error of the Mean (SEM). * p < 0.05. Where not indicated with *, differences are not significant. One-way ANOVA.

Immune cell infiltration to the inoculation site was determined by immunohistochemical staining of collected samples. Slides were stained with anti-CD8^+^ and anti-GrB antibodies to assess the presence of cytotoxic lymphocytes (Figure 5A).

Groups K7M2luc A7 and K7M2luc E5 exhibited the highest CD8^+^ infiltration, with 120 ± 17/10 HPF and 106 ± 16/10 HPF counted spots (Figure 5A,B), respectively. In contrast, the remaining two groups showed markedly lower CD8^+^ infiltration, with 20-35/10 HPF CD8^+^ spots counted (p = 0.0077 - 0.0285).

The highest number of GrB-positive cells (218 ± 2/10 HPF) was observed in the K7M2luc E5 group (Figure 5A,C) which was followed with a reduction (157 ± 9/10 HPF) in K7M2luc A7 group. K7M2 wt and Naïve groups showed the lowest GrB-positive cell counts (70 ± 11/10 HPF and 48 ± 3/10 HPF spots, respectively), which were lower than those observed in K7M2luc A7 (p < 0.0001, p < 0.0001). K7M2 wt and Naïve group were also lower than K7M2luc E5 group (p < 0.0001, p < 0.0001). Although differences were statistically significant, interpretation is limited due to small sample sizes.

### Establishment and growth curve monitoring of K7M2luc A7 cell line in NUDE mice

**FIGURE 6.**
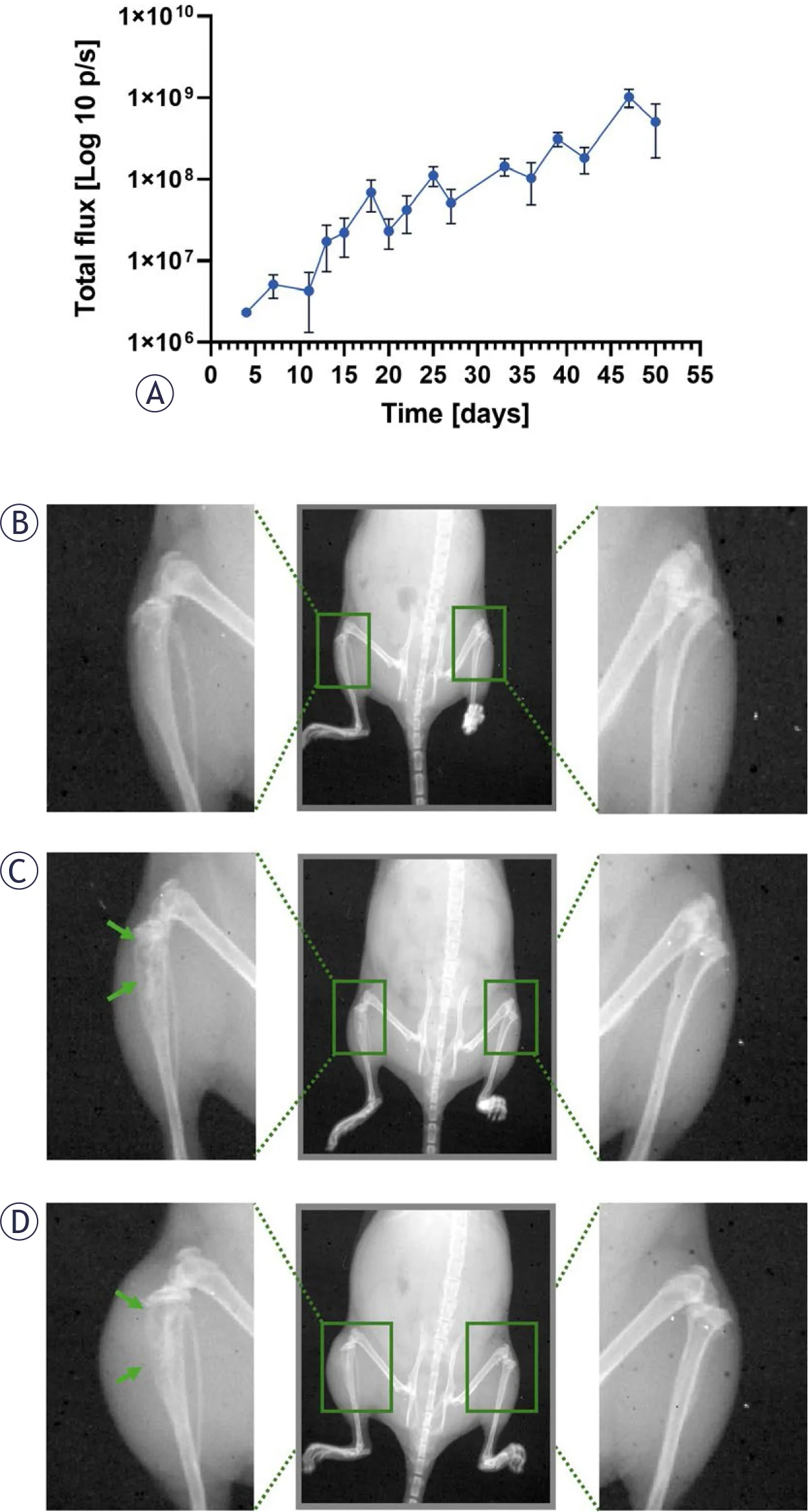
**(A)** Bioluminescent signal growth in NUDE mice, inoculated with K7M2luc A7 cells. **(B-D)** X-ray images of NUDE mice, inoculated with K7M2luc A7 cells on day 8, 25, 50. n = 4. Arrows mark bone lysis. Values are presented as Arithmetic Mean (AM) ± Standard Error of the Mean (SEM).

To confirm the premise of immunogenic properties of luciferase, six immunocompromised NUDE mice were inoculated with 1 × 10^6^ K7M2luc A7 cells. In all six mice establishments of the tumor was confirmed based on previous results from BALB/c mice. In those mice a drastic fall of signal strength on or after day 6 meant a subsequent failed tumor growth. Therefore, tumor establishment was assumed to be marked by stable signal above 1 × 10^6^ p/s at two consecutive BLI starting on day 7. For this reason, 2 mice were euthanized at the time of tumor establishment for histopathological evaluation and another 4 were monitored for further growth monitoring with BLI twice a week and X-ray imaging once weekly. First BLI was performed on day 4 post-inoculation (Figure 6A). From initial values of 2.3 × 10^6^ ± 9 × 10^4^ p/s the signal steadily rose until day 50 (5.9 × 10^8^ ± 3.3 × 10^8^ p/s). X-ray imaging showed intact bone on day 8 postinoculation (Figure 6B), indicating no damage due to surgical implantation of cells. Bone was intact up until day 25 (Figure 6C), when bone lysis was visible, demonstrating a progressive tumor growth. However, mice showed no clinical signs of distress up until day 50, when extensive bone lysis was visible and mice started to exhibit lack of grooming, minimal loss of weight and limping, at which point they were humanely euthanized (Figure 6D).

**FIGURE 7.**
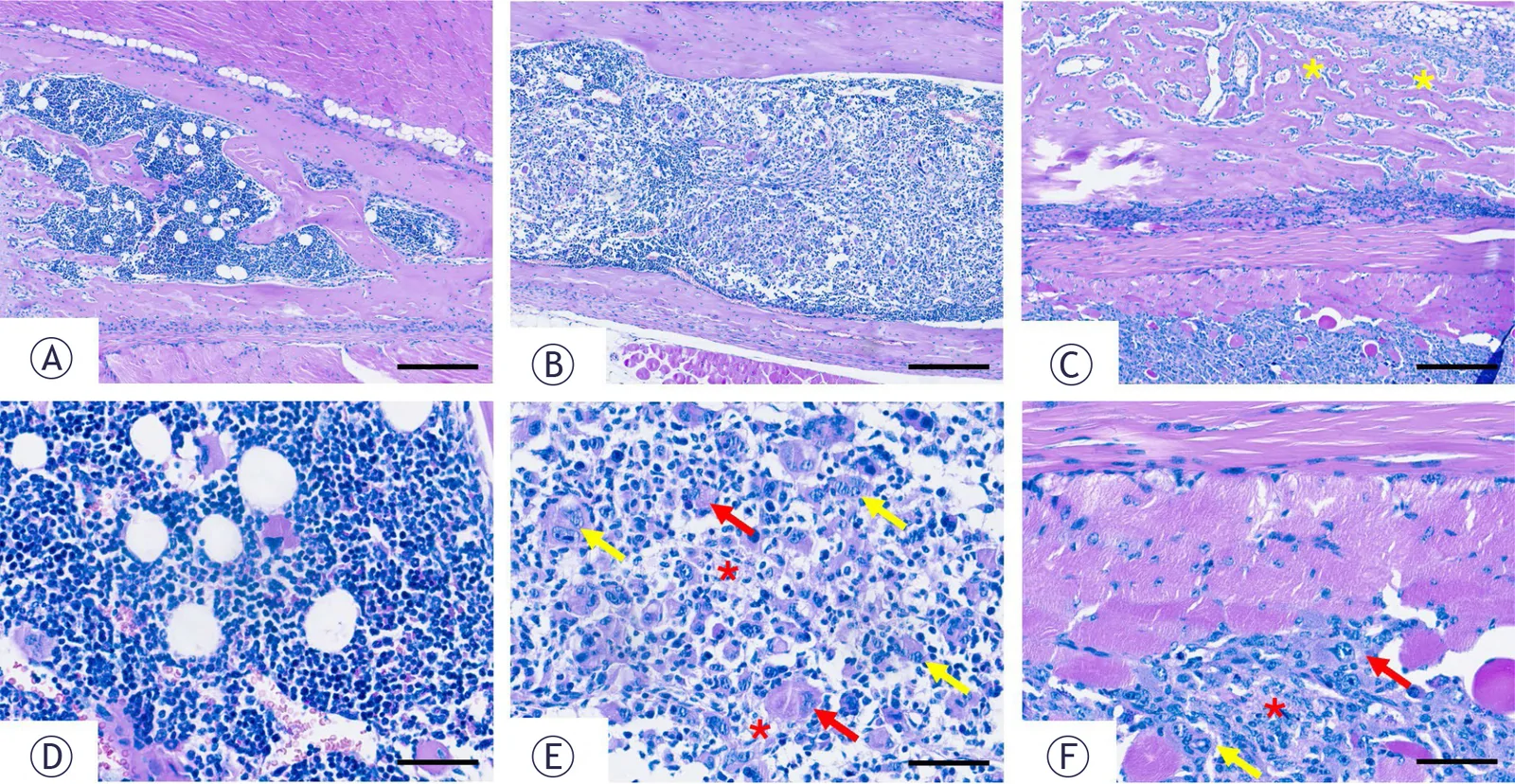
Representative images of paraffin tibia samples; **(A, D)** healthy, **(B, E)** established and **(C, F)** progressive tumor with soft tissue invasion. Yellow arrows mark tumor cells with enlarged or multiple nuclei, red arrows mark changed nucleus:cytoplasm ratio in tumor cells, red asterisks mark lace-like malignant osteoid matrix and yellow asterisks mark bone lysis. **(A, D)** 5x magnification, scale bar = 100 μm. **(B, C, E, F)** 40x magnification, scale bar = 60 μm.

The histopathological evaluation of two euthanized mice at the time of tumor establishment showed typical pathological changes after K7M2luc inoculation when comparing healthy (Figure 7A,D) and tumor-affected (Figure 7B,E) bone histological sections. Differences included presence of viable tumor cells with enlarged or multiple nuclei (Figure 7E-F: yellow arrows), changes in nucleus:cytoplasm ratio (Figure 7E-F: red arrows) and lace-like malignant osteoid matrix formation (Figure 7E-F: red asterisks). Furthermore, samples taken on day 30 show signs of progressive disease (Figure 7C,F), where bone lysis is visible (Figure 7C: yellow asterisks) and tumor cells invade the surrounding soft tissue (Figure 7F).

## Discussion

The aim of this study was to evaluate immunogenic changes after orthotopic implantation of K7M2 luciferase-expressing cells into mouse tibia. One of the main characteristics of osteosarcoma tumors is their microenvironment, therefore there is a need for an orthotopic tumor model as opposed to a more widely used subcutaneous one. Furthermore, to evaluate antitumor effectiveness of novel therapies on orthotopic tumor models, *in vivo* imaging should be employed.^38^

In this study, we successfully transduced mouse osteosarcoma cell line K7M2 wt with luciferase gene using in-house developed plasmid and commercially available viral particles. (K7M2luc A7, K7M2luc E5). When selecting clonal cell lines, clones exhibiting sufficient bioluminescent intensity were chosen, favoring high, but not maximal, expression levels, as excessively high expression has been associated with reduced *in vivo* tumor growth.^22^

Despite slightly shorter doubling time observed in K7M2luc E5 cell line, no statistically significant differences in doubling time were found between wt and luciferase-expressing cell lines. This indicates that luciferase expression does not alter *in vitro* cell proliferation, which was previously also reported by Ferrari *et al*., 2024.^20^ Next, we orthotopically implanted these two cell lines to syngeneic BALB/c mice. The results of *in vivo* orthotopic model showed there were differences between luciferase-expressing cell lines and wt. Although luciferase-expressing cells produced a detectable bioluminescent signal at the BLI on day 3, signal rapidly declined to below detection threshold (1 × 10^4^ p/s). As a result, inoculation success rate was 0%, which contrasts with previously reported rates of 80% and 90% using similar luciferase-tagged K7M2 lines.^39^ Using K7M2 wt cell line, our inoculation rate was 100%, which is consistent with literature, where they reported an inoculation rate of 92%.^39^

One possibility for unsuccessful tumor establishment could be attributed to the immunogenic characteristics of luciferase. There have been several papers published describing induced immunogenicity, which negatively affected the growth of KPC tumors in pancreatic ductal adenocarcinoma models as well as in case of orthotopic 4T1 tumors, where unsuccessful implantation of the tumor in the mammary fat pad was attributed to the development of IFN-γ response.^20,25^

Therefore, we examined possible effects of luciferase on inducing antitumor immune response. We focused on investigating cell-mediated immunity, which is activated by intracellular changes and eliminates abnormal cells, including tumor cells.^40^ Luciferase as a novel protein expressed in tumor cells supposedly presents as a foreign antigen, which is detected by immune system.^21,25,27^ Humoral response, targeting extracellular pathogens, was already proven in previous reports to be inactive in the presence of luciferase.^20^ NK cells and CD8^+^ cells are leading anticancer immune responders, causing tumor suppression by releasing cytokines, granzymes and other effector molecules.^41^ Activation of spleen-derived cytotoxic immune cells was tested via GrB and IFN-γ secretion. Luciferase-expressing cells showed an activation of cytotoxic lymphocytes, leading to elevated GrB secretion in comparison to samples from naïve mice. This was similar to KPC-luc spleen samples where a significant increase in NK T cell presence was reported, as well as in activated CD8^+^ cells, expressing IFN-γ.^20^

Additionally, we evaluated tumor inoculation site for the presence of immune cells that would be accounted for the local immune response and rejection of the tumor. Immunohistochemical staining for GrB showed increase in K7M2luc samples compared to both wt and naïve mice, indicating presence of activated cytotoxic lymphocytes. Moreover, K7M2luc E5 had a higher number of GrB positive cells in comparison to K7M2luc A7. Such results indicate K7M2luc E5 as being more immunogenic, probably due to a higher luciferase expression, which was observed *in vitro*. Similarly other groups already reported a connection between higher luciferase expression in a cell line and reduced tumor growth *in vivo*.^22^ In order to determine whether GrB is secreted from activated CD8^+^ or NK cells, we also stained for CD8^+^ cells. Both K7M2luc exhibited a significantly higher concentration of CD8^+^ cells as opposed to wt and naïve samples. It can be thus supposed that tumor regression mainly occurs due to localized presence of activated CD8^+^ cytotoxic cells with limited systemic cytokine elevation. Owyang *et al*. published similar conclusions, where no systemic anti-tumor reactivity was detected in murine colon carcinoma CT26 tumors and tumor regression was limited to changes in TME.^42^ Therefore, despite vast usage of luciferase and other imaging markers, considerations must be made as to the possible detrimental impact on tumor model development.

To further confirm the involvement of cytotoxic T lymphocytes in tumor establishment of K7M2luc orthotopic tumors, we inoculated NUDE mice with only K7M2luc A7 cells since K7M2luc E5 cells demonstrated higher immunogenicity, likely associated with their increased luciferase expression observed *in vitro*. Similar associations between high luciferase expression and reduced *in vivo* tumor growth have previously been reported.^22^ Immunodeficient NUDE mice lack T lymphocytes, such as CD8^+^, but still contain innate immune components like NK cells that could act as anti-tumor effectors.^43^ Results from histological analysis of tumors in NUDE mice confirmed the chosen criteria for tumor establishment, which was as followed: starting 6 days post inoculation two consecutive BLI signal strengths hold above 1 × 10^6^ p/s. Histochemical staining of samples, taken at the point of tumor establishment confirmed presence of tumor cells and changes in bone tissue, consistent with murine OSA histopathological markers.^44-47^ Following tumor growth by BLI showed that tumor establishment occurred 7-11 days after tumor implantation. Further on, on day 15, signal strength was between 2.31 × 10^6^ and 4.13× 10^7^ p/s, which is comparable to values stated by Grisez *et al*. at day 14 (approximately 2.5 × 10^6^ p/s). They reported further stable signal growth with a peak on day 21 of approximately 1.5 × 10^7^ p/s and subsequent tumor signal stagnation, whereas our model displayed a stable, progressive growth until day 50.^26^

Staining for GrB-secreting and CD8^+^ cells revealed discrepancies in the number of positively stained cells, with a higher abundance of GrB-secreting cells observed. This difference may be attributable to the presence of NK cells secreting GrB. Hypothesis, that adaptive immunity represents the primary mechanism underlying tumor establishment failure, was based on previous reports.^20^ Successful tumor establishment and further stable growth as visible by BLI and X-ray imaging confirmed that although NK cells may be present and could act as tumor suppressors, they are not sufficient for tumor rejection in case of OSA luciferase-expressing orthotopic tumors. Presence of activated CD8^+^ is crucial for complete tumor regression, which was already shown in our previous studies, where activation of innate immune system effectors such as NK cells in NUDE mice led to tumor growth delay, whereas in immunocompetent mice both arms (NK cells and CD8^+^ cells) of antitumor immune response were activated and succeeded in complete eradication of the tumor.^48^ Our results are also in line with other studies, where they observed faster growth of adaptive immune system inhibiting CT26-TGF-ß-R tumors in NUDE mice as opposed to immunocompetent mice.^42^ Furthermore, another murine colon adenocarcinoma tumor model CT-26 expressing luciferase exhibited differences in tumor growth between immunocompetent and immunodeficient mice. A notable tumor growth retardation was observed in immunocompetent mice, but not in immunodeficient.^49^

In conclusion, our study demonstrated that luciferase-induced immune responses in syngeneic mice interfered with the establishment of orthotopic osteosarcoma tumors, necessitating the use of NUDE mice. However, immunocompromised models present certain challenges, particularly for evaluation of antitumor effect of novel immunotherapies.

Some limitations of this study should be acknowledged. First, the ELISpot assay did not include an exogenous antigen-specific positive control, which limits direct comparison of the observed responses with well-characterized immunodominant epitopes. However, the assay was intended to assess relative differences in cytotoxic responses across experimental groups under identical conditions. Therefore, conclusions are based on within-assay comparisons rather than absolute benchmarks of antigen-specific immunogenicity. Furthermore, luciferase-specific immune responses were evaluated using only a single luciferase-derived peptide, which may not fully reflect the range of antigenic epitopes generated from luciferase expression *in vivo*. Use of an entire luc2 peptide pool would allow for a more comprehensive evaluation of luciferase-specific cytotoxic responses. Another limitation was the inability to detect IFN-γ in either serum or supernatant samples, despite secondary in vitro stimulation of splenocytes using a peptide containing the immunodominant luciferase-derived CD8+ epitope in the BALB/c background.^33^ These findings contrast with previously published reports demonstrating elevated serum IFN-γ levels in mice bearing luciferaseexpressing tumors compared with wt tumors.^25^ Several factors may account for this discrepancy. Firstly, IFN-γ production is highly dynamic and often transient during antitumor immune responses. Because samples in our study were collected at experimental endpoints, it is possible that peak cytokine production had already passed by the time of analysis.^50^ Another reason for lack of detection could be that the levels produced were below the threshold of our tests. In addition, a limitation of this study is the low number of samples, which reduces statistical robustness. In line with the 3R principles, the results were derived from a study comprising three mice per group, which further limits the strength and generalizability of the conclusions.

Nonetheless, these findings should further raise awareness of this critical yet often overlooked experimental variable and encourage the use and development of alternative models for preclinical cancer research. One such example are luciferase-tolerant transgenic strains (Tol mice). Other possibilities include alternative reporter systems with lower immunogenic potential, like click green beetle luciferase, which reportedly has minimal immunogenic effects in C57BL/6 mice. Precautions can also be taken in preparation of clones of parental cell lines, that express lower levels of luciferase that can still be detected.^21,22,51^
